# Low-Intensity Focused Ultrasound Alters Alzheimer’s Disease Pathology, In Vivo, as a Function of Ultrasound Dose and Age

**DOI:** 10.3390/brainsci16070757

**Published:** 2026-07-18

**Authors:** Alissa Phutirat, Kahte A. Culevski, Hannah Mach, Jamie Kwon, Henry Tan, Gabe Koh, Caren Marzban, Pierre D. Mourad

**Affiliations:** 1 Department of Neurological Surgery, University of Washington, Seattle, WA 98195, USA; alissaphutirat@gmail.com (A.P.); happykittykahte@gmail.com (K.A.C.); hannahpm@uw.edu (H.M.); jkwon4@stanford.edu (J.K.); henrytan@uw.edu (H.T.); gkoh01@uw.edu (G.K.); 2 Applied Physics Laboratory, University of Washington, Seattle, WA 98195, USA; marzban@uw.edu; 3 Division of Engineering and Mathematics, University of Washington, Bothell, WA 98011, USA

**Keywords:** Alzheimer’s disease, vascular dementia, focused ultrasound, FUS

## Abstract

Background/Objectives: Alzheimer’s Disease (AD) and vascular dementia contribute up to ~75% of dementia cases, as determined via autopsy. AD arises in part due to the buildup of aberrant proteins (amyloid beta (Aβ) and Tau); vascular dementia is caused by reduced cerebral blood flow. Each dementia mechanisms damages brain. Bobola et al. found that their low-intensity focused ultrasound (FUS) protocol applied to the brains of the 5XFAD mouse model of AD reduced Aβ by 50% through activation of microglia. Eguchi et al. found that their own FUS protocol applied to the brains of the same mouse model reduced Aβ by 15% and increased cerebral blood flow by 50% through an increase in endothelial nitric oxide synthase (eNOS). Here, we sought to test a combined version of those two FUS protocols, expecting both a decrease in Aβ burden and an increase in eNOS. Methods: Using a diagnostic ultrasound probe, we applied our combined FUS protocol primarily to the left hippocampus of anesthetized 5XFAD mice, for an hour a day, for three days for younger mice and for five days for older mice. On day three or five, respectively, we harvested their brains and performed histological analysis to assess Aβ burden, microglial activation and their co-localization with Aβ, as well as the burden of eNOS within neuronal nuclei (here called intra-neuronal eNOS) and outside of neurons. Results: Relative to untreated mice, the treated younger mice had more activated microglia co-localized with Aβ and reduced Aβ burden for large plaques, as well as no change in each measure of eNOS. In contrast, the treated older AD mice had no change in activated microglia co-localized with Aβ, and no change in Aβ burden. However, relative to untreated older AD mice, FUS decreased total and extra-neuronal eNOS and increased intra-neuronal eNOS. Conclusions: The ability of our FUS protocol to reduce Aβ burden and alter the eNOS distribution depends critically upon the age of the AD mice (more Aβ plaques for a comparable number of microglia for older mice relative to younger mice) and duration of the treatment. The observed decrease in extra-neuronal eNOS distribution in older AD mice caused by FUS raises the concern that our protocol may increase ischemia, while the increase in intra-neuronal eNOS may counteract that effect via protection of synaptic function. These findings also identify two candidate therapeutic windows for our FUS treatment protocol, each requiring more research before translation to humans. One window is early intervention to maximize Aβ plaque removal via activation of microglia. The second is later intervention to protect synaptic function if it is possible to mitigate the potential ischemic risk caused by the differential effects of FUS on eNOS.

## 1. Introduction

Dementia is a class of devastating neurodegenerative disorders with a huge personal and societal burden [1]. A prominent form known as Alzheimer’s Disease (AD) is characterized histologically by the accumulation of amyloid-β (Aβ) plaques and phosphorylated Tau tangles (p-Tau) [2,3,4] and clinically by profound cognitive decline, progressive memory loss, and early mortality [5]. The dominant view of AD identifies the accumulation of those aberrant proteins, especially p-Tau, as playing a causal role in the progression and effects of this disease, a complex topic with a range of studies supporting or refuting that view [6]. Pure vascular dementia—a reduction in cerebral blood flow correlated with aging—also damages the human brain in the long term [5]. Individually, AD and vascular dementia account for ~20% of dementia cases, while their combination (mixed dementia) accounts for ~50% of dementia cases, as determined by autopsy [5].

There exists a role for nitric oxide synthase (NOS) in vascular, AD, and mixed dementia. NOS is expressed predominantly in endothelial cells (known as eNOS), where it helps regulate vascular tone and hence cerebral blood flow. eNOS is also expressed in the nuclei of neurons, where in the context of AD, it can maintain synaptic function during a significant percentage of progression of the disease [7]. Nonetheless, aberrant nitric oxide presence and function can contribute significantly to a variety of neurodegenerative processes, including necrosis, apoptosis, autophagy, and neuronal death, all relevant to Alzheimer’s Disease pathogenesis [8,9].

Recently approved medications (Donanemab and Lecanemab) have discernable though modest impact on the rate of cognitive decline through a reduction in Aβ plaque burden [10]. Among the variety of therapeutic alternatives to medications, recent research has highlighted the potential of non-pharmacological approaches to reduce the burden of Aβ plaque and Tau. Light-based stimulation of AD brain with frequencies in the gamma band, specifically 40 Hz, cleared ~50% of Aβ from the visual cortex of awake 5XFAD mice exposed to five days of one-hour sessions of 40 Hz blinking light relative to sham [11]. Subsequent work combining light and acoustic stimulation in this frequency band showed reduction in Aβ in the visual and auditory cortices, as well as the prefrontal cortex and hippocampus of 5XFAD mice [12]. This remains an area of active research [13]. Enhancing gamma activity in the brain is an a priori plausible approach to treating dementia because gamma oscillations participate in perception and cognitive function, with impairments in this frequency band in AD mouse models and patients (reviewed by [11]).

Focused ultrasound (FUS) with microbubbles induces localized mechanical behavior of the microbubbles that disrupts the blood–brain barrier (BBB). This is a promising and very active area of research. Leinenga and Götz [14] presented an early example of this approach. They demonstrated that four FUS sessions distributed over eight weeks reduced the Aβ plaque burden and improved the memory of a mouse model of AD, and this was followed up by Karakatsani et al. [15]. Recently, Rezai et al. [16] demonstrated that the same procedure applied to AD patients increased the flux of an FDA-cleared antibody against Aβ plaques, safely producing a significant reduction in Aβ plaque burden relative to contralateral brain tissue not exposed to FUS. Mehta et al. [17] offer a review of this topic.

FUS that does not disrupt the BBB can also improve dementia pathology and/or symptoms. The original FUS protocol without microbubbles used by Götz and colleagues did not alter AD pathology [18]. Their application of a different FUS protocol has improved AD symptoms, in vivo, with and without alteration in the underlying aberrant protein burden—as reviewed in Balbi et al. [19]. Human studies of FUS for dementia are emerging, such as Nicodemus et al.’s [20]. Motivated by the 40 Hz pulsed-light treatment of AD pathology cited above, Bobola et al. [21] demonstrated comparable microglial activation acutely and comparable (~50%) and rapid (over five days) reduction in Aβ plaques, also in 5XFAD mice. They used 40 Hz pulses of transcranial, focused ultrasound without microbubbles to disrupt the BBB. Importantly, this occurred in and around the targeted hippocampus, rather than only in the visual cortex, as reported by [11]. Park et al. [22] corroborated these observations and extended them, observing changes in brain connectivity and enhancement in spontaneous EEG gamma power. Yet others have taken a separate approach to reducing Aβ plaque burdens in an anesthetized 5XFAD mouse model without concurrent use of microbubbles (Eguchi et al. [23]). Their FUS protocol, different from that by Bobola et al. [21] and Park et al. [22], causally increased total eNOS protein expression and cerebral blood flow and reduced Aβ plaques after a three-session-per-week, two-month treatment protocol.

Given the importance of pure AD, vascular dementia, and their combination, we constructed a protocol that combines the temporal patterns of each of Bobola et al.’s [21] and Eguchi et al.’s [23], thereby seeking to produce a combination of their therapeutic effects in AD mouse brain. We first applied that protocol to relatively younger AD mice, for three days. Those results motivated subsequent application to a substantially older cohort, for five days. The biological results depended strongly on the intervention’s duration in terms of days as well as mouse age.

## 2. Materials and Methods

### 2.1. Overview of Experimental Procedures

#### 2.1.1. Animal Model

We used female 5XFAD (C57BL/6) transgenic mice (MMRRC stock #34840; Jackson Laboratory, Bar Harbor, ME, USA), randomly distributed between sham- and FUS-treated cohorts. (Female 5XFAD mice exhibit more pathology than male mice.) This model overexpresses mutant human APP(695) with the Swedish (K670 N/M671L), Florida (I716V), and London (V717I) Familial AD (FAD) mutations and human PS1 with two FAD mutations, M146L and L286V [24]. 5XFAD (C57BL/6) mice present with a significant Aβ plaque burden that emerges between 2 and 4 months of age. The relatively ‘younger’ cohort of 5XFAD mice we used had a median age of 7.5 +/− 0.51 months and experienced three days of FUS therapy. The relatively old mice—here called ‘older’ mice—had a median age of 12.17 +/− 0.13 months and experienced five days of FUS therapy. The extent of pathological and functional change increases as 5XFAD mice mature [25]. For example, at 9–12 months of age, 5XFAD mice fully display parenchymal and vascular Aβ plaques, synaptic and neuronal loss, neuroinflammation, and gliosis, as well as motor and cognitive deficits, compared with their wild type (WT). In contrast, 5XFAD mice at 7 months of age have Aβ plaques in multiple brain areas, activated microglia, and dystrophic neurites, though without demonstrating synaptic and neuronal loss. This difference in pathology between these age groups prompted us to apply our FUS therapy to two cohorts of mice that differ in age and then for different lengths of FUS therapy. Interestingly, according to reference [26], the younger 5XFAD mice correspond to ~30 year-old humans, while the older 5XFAD mice correspond to ~42 year-old humans; however, the pathology (and functional) deficits experienced by these mice correspond to much older humans.

We used G* power version 3.1.9.6 [27] and the results obtained by Bobola et al. (47 +/− 8% reduction in Aβ plaques due to FUS relative to sham), along with the assumptions of alpha = 0.05 and beta = 95%, to support our use of n = 3 mice in each of sham and treated cohorts in our study.

#### 2.1.2. Timeline of Three-Day Chronic Study

**Figure 1:**
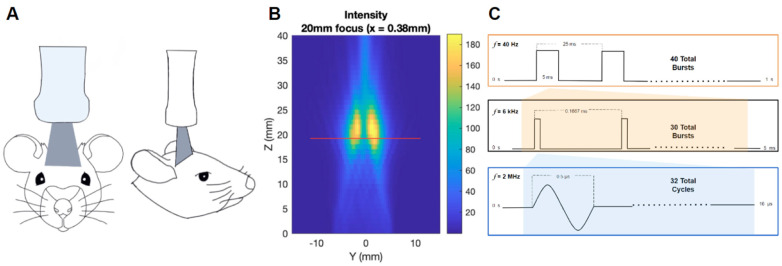
(**A**) Position of ultrasound relative to bregma on a mouse skull, viewed from two orientations. (**B**) Holographic image of the FUS beam in the radial (y) plane as a function of distance from the probe face. The red line denotes where we measured the intensity value (Isppa = 90 W/cm^2^) at the left focus, ~20% more intense than at the right focus—the target of our histological analysis. (**C**) Schematic of our novel FUS protocol.

We (sham-)treated three anesthetized younger 5XFAD mice with FUS applied simultaneously to each hemisphere of their brains (Figure 1A), though more in the left than right hemisphere as described below, for one hour per day, for two days. On the third day, after re-establishing an anesthetic plane, electrocorticography (ECoG) wires were placed in the hippocampus and somatosensory cortex within each brain hemisphere to measure brain activation in response to one more hour of FUS. We then sacrificed and perfused the mice. An additional three mice were exposed to sham FUS under the same pre/post-ultrasound procedures.

#### 2.1.3. Timeline of Five-Day Chronic Study

We (sham-)treated three anesthetized older 5XFAD mice with FUS applied simultaneously to each hemisphere of their brains (Figure 1A), though more in the left than right hemisphere as described below, for one hour per day, for four days. On the fifth day, after re-establishing an anesthetic plane, ECoG wires were placed in the hippocampus and somatosensory cortex within each brain hemisphere to measure brain activation in response to an additional dose of FUS for one hour. We then sacrificed and perfused the mice. An additional three mice were exposed to sham FUS under the same pre/post-ultrasound procedures.

### 2.2. Detail of Experimental Procedures

#### 2.2.1. Anesthesia

After initial exposure to isoflurane at 2–3% and placement within the experimental setup (Figure 1A), mice remained exposed to isoflurane at 1.5–2.5% through a nose cone to maintain sedation throughout the experiment, adjusted as necessary to keep a healthy anesthetic plane.

#### 2.2.2. Experimental Setup—Chronic Studies Without ECoG

After induction of an anesthetic plane using isoflurane, we placed the treatment animal on a heating pad and into a mouse stereotaxic headpiece (Stoelting Digital Lab Standard, Wood Dale, IL, USA). We then applied ophthalmic ointment (Artificial Tears; Akorn; Gurnee, IL, USA) to their eyes followed by a depilatory cream (Nair Hair Remover Cocoa Butter; Nair; Ewing, NJ, USA) to the top of the mouse head in order to remove its fur. We attached a custom, 3D-printed pointer to the distal face of the diagnostic ultrasound transducer to provide a visual cue for the focal ultrasound projected by that transducer. The pointer plus our digital stereotax guidance system (WPI, Sarasota, FL, USA) allowed us to guide the ultrasound transducer over the correct portion of the head of the mouse. We then placed ultrasound gel (Aquasonic 100 Ultrasonic Gel; Next Level Technology; Los Angeles, CA, USA) on the dorsal portion of the mouse’s head, lowered the distal face of the transducer into the gel, then applied FUS (Figure 1A). Specifically, we placed the FUS focus at 1.43 mm posterior of Bregma and 1.3 mm below the skin surface, into sections C1–C3 of the hippocampus and surrounding brain tissue.

#### 2.2.3. Experimental Setup—Acute Study with ECoG

After induction of an anesthetic plane and removing hair with Nair as above, we injected lidocaine and bupivacaine (AuroMedics Pharma; East Windsor, NJ, USA) subcutaneously into the dorsal aspect of the mouse skull, surgically exposed the skull, and then drilled holes for placement of electrodes in the motor cortex and hippocampus of each hemisphere, with coordinates determined by the Allen Institute’s online mouse atlas. We created custom-made ECoG electrode arrays by soldering five silver electrode wires (0.0130-inch coated/0.010-inch bare diameter), as well as one silver ground wire (0.0190-inch coated/0.015-inch bare diameter). We connected that array into a preamplifier chip, itself connected to a biosignal analog converter (Pinnacle Technology, Lawrence, KS, USA). ECoG signals propagated from the chip into PowerLab, whence into LabChart Version 8 (AD Instruments Inc.; Dunedin, New Zealand), for the real-time monitoring of the data and to facilitate post hoc analysis with MATLAB Version R2023a (MathWorks, Inc., Natick, MA, USA).

#### 2.2.4. Ultrasound Setup and Protocol

We used a Vantage™ V-1 Research Ultrasound System (Verasonics Inc.; Kirkland, WA, USA) with a P42 diagnostic ultrasound probe (ATL Ultrasound, Philips Medical; Bothell, WA, USA). The spatial peak pulse average (Isppa) was 90 W/cm^2^ (less than half the value used by Bobola et al. [21] and below the diagnostic ultrasound limit on Isppa), as measured in water at a distance where it would overlap with the hippocampus (Figure 1B). The acoustic output of the P-42 transducer was measured using an Onda HNR-500 needle hydrophone (Sunnyvale, CA, USA). Prior to data collection, water in the scanning tank was degassed to less than 2% dissolved oxygen. A series of 1D and 2D scans were collected to characterize the acoustic sound field at low pressures and extrapolated to higher pressures (Figure 1B). Additional measurements were made away from the focus at higher voltages to confirm linearity (pressure as a function of voltage) of the Verasonics power supply and confirm that the Verasonics could output long-duration pulses as used in this study without droop. The FUS protocol (Figure 1C) combined the large-scale pulse pattern used by Bobola et al. [21] with the fine-scale pulse pattern of Eguchi et al.’s study [23]. This protocol used a carrier frequency of 2 MHz, pulsed 40 times/s (40 Hz PRF), each lasting for 5 ms. Within each 5 ms long pulse, we distributed 30 bursts that lasted for 400 μs each, delivered at a rate of 6 kHz. Each session lasted one hour.

#### 2.2.5. Euthanasia and Brain Harvesting

Deeply anesthetized mice were immediately euthanized after the final FUS treatment using the transcardiac perfusion method with formaldehyde in order to preserve brain tissue. Brains were promptly and carefully extracted from the skull and stored in formalin.

#### 2.2.6. Histology

Brain tissue was processed by the Fred Hutchinson Department of Histopathology. We used the left hemisphere for all our histopathology analysis because it was exposed to more FUS than the right hemisphere, which is also why we had a separate sham group rather than offering inter-hemispheric comparisons within the same mouse. Also, due to loss of skilled personnel during to the COVID-19 pandemic, we were able to use fluorescent imaging for brain tissue only for mice from the three-day chronic study. We had to switch to DAB staining for microglia, Aβ and eNOS plus Eosin for cell nuclei for mice for the five-day chronic study.

Mouse brains were fixed in NFB (neutral buffered formalin) through perfusion, trimmed to the site of ultrasound application and sent to our vendor in 70% ethanol. Samples were then processed in paraffin blocks and sliced at the region of interest in 12 serial slices per brain with two slices of tissue placed per slide. The ROI began at the center of the ultrasound focus and then proceeded rostral in consecutive slices. For the three-day chronic study, each slice measured four microns in thickness. The slides were stained from the center of the US application to the rostral portion in this order: two slides with a total for four slices stained for Aβ + microglia + DAPI, two slides with a total for four slices stained for Aβ + eNOS + DAPI, two slides with a total for four slices stained for Hematoxylin and Eosin. For the five-day chronic study, each slice measured 7–10 microns in thickness. The slides were stained from the center of the US application to the rostral portion in this order: one slide with two slices stained for Aβ, one slide with two slices stained for eNOS and Eosin.

Aβ was stained with rabbit monoclonal anti-beta amyloid 1–42 antibody (Clone mOC64; Abcam, Cambridge, UK, Cat. No. Ab201060). Microglia were stained with rabbit polyclonal anti-Iba1 (Wako Chem, Osaka, Japan; Cat. NO. 019-19741). eNOS was stained with Rabbit anti Phospho-eNOS antibodies known to bind to activated eNOS (Antibodies-Online Inc., Cat. No. ABIN6256115; Limerick, PA, USA). For fluorescent imaging, we stained microglia with Alexa Fluor 488 Dye, Aβ with Cy3 Dye and nuclei with Invitrogen DAPI counterstain (4′,6-diamidino-2-phenylindole, dihydrochloride; Cat. # D1306), each from ThermoFisher Scientific (Waltham, MA, USA).

### 2.3. Overview of Image Analysis

#### 2.3.1. Image Acquisition

Images of histological tissue slides from the left side of each mouse brain were captured at 125× magnification using ZEISS ZEN 3.6 (blue edition) microscopy software and an immunofluorescent microscope (Zeiss Axiozoom V.16; Oberkochen, Baden-Württemberg, Germany). For standardization, the eNOS channel in far red was set as the reference channel. Green and blue channels were added for detecting Aβ and nuclei, respectively. Using a range indicator, intensity of the image was adjusted, and exposures noted. Images were saved and exported as a tri-channel CZI file.

#### 2.3.2. Image Analysis

We processed histopathological images using FIJI (version 2.9.0), a free image processing package within ImageJ (version 1.53). Images were uploaded with the most pixels to reflect the realistic dimensions of the brain slice based on length-per-pixel measurements. We performed our analysis within the left hemisphere of each slide, consistent with the placement of the more intense FUS focus. Using the polygon selection, the outline of the brain was traced as a region of interest (ROI). If they were visible, the ventricles were also outlined then subtracted from the larger ROI. The images were then filtered to remove possible artifacts by shape and size. This included running a macro that standardized a scale of 0.93 µm per pixel. We identified common structures in adjacent slices to align features of interest for subsequent analysis. All images had standardized filtering which consisted of running a ‘Subtract Background’ with a ‘Rolling ball radius’ of 50.0 pixels and the light option. The option ‘Otsu’ was used to create a binary. The ‘Extended Particle Analyzer’ from BioVoxxel Toolbox was used to analyze microglia, Aβ and nuclei. The microglia particles were identified by those having an area greater than 78 µm^2^. Microglia identified as active had a roundness factor between 0.523 and 1.0 (Bobola et al. [21]).

#### 2.3.3. Co-Localization of Active Microglia and Plaques

Both microglia and Aβ plaques were made binary and counted using the particle analyzer in FIJI, through which size, roundness, location, and area were obtained. Inactive microglia were characterized by identification of their long and thin skeletal bodies, whereas active microglia were characterized by identification of their hypertrophic cell body and more retracted processes. Activated microglia in the images were formally identified as having a sufficiently ‘round’ structure—quantitatively identified by the roundness function with a minimum value of 0.523 as in Bobola et al. [21]; see also their Figure 2. Plaques that had at least one active microglia overlapping within 0.15 µm were considered co-localized, as in Bobola et al. [21]. The percentage of plaques co-localized with microglia were calculated within each tile across a comparably sized region of interest between sham- and FUS-treated mice.

#### 2.3.4. Plaque Burden

After each plaque was accounted for, we created quantile–quantile plots to determine the range of plaque sizes for which there existed a difference in population. A size range by area (in square microns—µm^2^) was thus created for mice from each cohort. The percentage of plaques, including as a function of individual plaque area, was calculated within each tile across a comparably sized region of interest between sham- and FUS-treated mice.

#### 2.3.5. Quantile–Quantile Analysis

This technique determines if two data sets—one graphed along each of the horizontal and vertical axes—come from populations with a common distribution. Data points along a 45-degree line (blue in the figures) come from the same distribution. Those data points that deviate meaningfully from that 45-degree line may come from a different distribution, thus motivating a formal statistical test of that possibility. The red-dotted line shown on the graph results from a linear regression through the lowest-value data points. MATLAB was utilized to process histological Aβ plaque data and create quantile–quantile plots. Points at which the plaque size differed between two groups were noted using MATLAB and further analyzed with a Welch two-sample *t*-test [28].

#### 2.3.6. Quantification of eNOS

The percentage area of all eNOS was calculated within each tile across a comparably sized region of interest between sham- and FUS-treated mice. eNOS within nuclei of any cell were identified by its co-localization with Eosin, as appropriate. To differentiate between the nuclei of neurons versus endothelial cells, we noted differences in cell shape: neurons with many branches and complex shapes versus endothelial cells, which surround blood-vessel walls. We also noted differences in the circularity of the nuclei: nuclei in neurons are rounder than in endothelial cells. Regarding the latter point, the nuclei of neurons had a reference circularity filtering of 0.80–1.0, and only eNOS that overlapped with nuclei was counted as eNOS within the neuron [29,30].

#### 2.3.7. Statistical Analysis

Since there were two brain-tissue slices per slide, we used the Welch two-sample *t*-test in R software to assess any difference between sham and treated brains, co-localization, Aβ burden, and eNOS distribution [28]. For multiple comparisons, we first used ANOVA and then the Welch two-sample *t*-test when appropriate. We describe all results in terms of median +/− the standard error of the data.

## 3. Results

### 3.1. Histopathology and ECoG Results

**Figure 2:**
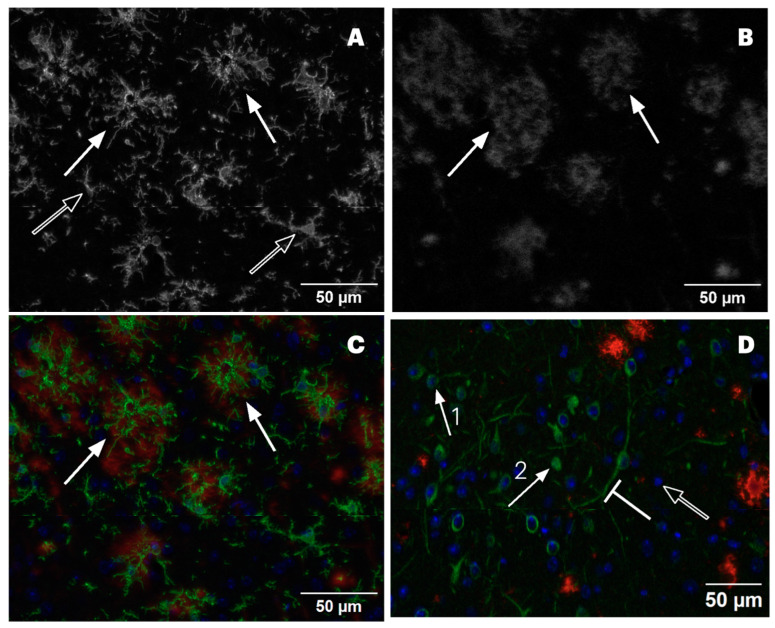
Representative immunofluorescent histopathology images from three-day-FUS-treated younger AD mice. (**A**) Microglia immunofluorescence image; closed arrows point to activated microglia; open arrows show inactivated microglia. (**B**) Closed arrows point to examples of Aβ plaques. (**C**) Closed arrows identify superposition of microglia, and Aβ plaque fluorescent images show overlap of activated microglia and Aβ plaques. (**D**) eNOS (green), nuclei (blue), and Aβ plaques (red). Here, closed arrow #1 points to eNOS within the nuclei of a neuron; closed arrow #2 and the bar-headed arrow point to eNOS outside of the nuclei of a neuron; the open arrow points to a cell nucleus without eNOS.

Figure 2 shows sample histology for Aβ plaques, microglia, and eNOS from the three-day, younger-AD-mouse study. Images offer examples of active and inactivated microglia, of their co-localization with Aβ plaques, and the presence of eNOS outside of and within neurons.

**Figure 3:**
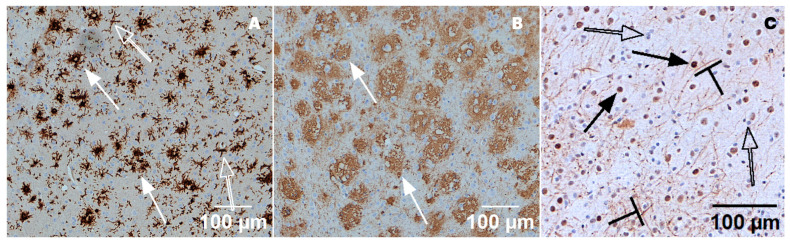
Representative histopathology image from five-day-FUS-treated older AD mice. (**A**) Activated microglia identified with closed arrows; inactivated microglia identified with open arrows. (**B**) An image of Aβ plaques taken from a slice of brain adjacent to the one shown in Figure 3A, with the closed arrows here corresponding to the closed arrows in Figure 3A. (**C**) An image of eNOS (brown) and cell nuclei (blue). The closed arrows point to intraneuronal eNOS overlapping with round neuronal nuclei; the open arrow points to a cell nucleus without eNOS; the bar-headed arrows show eNOS outside of nuclei.

Figure 3 shows sample histology for amyloid beta, microglia, and eNOS from the five-day, older-AD-mouse study. Images offer examples of active and inactivated microglia, of their co-localization with Aβ plaques, and the presence of eNOS outside of and within neurons.

In both the 3-day, younger cohort and the 5-day, older cohort, we observed co-localization of activated microglia and Aβ plaques. These results are consistent with our previous observations: Figure 2 in Bobola et al.’s study [21] shows individual microglia, and Figure 3 in the same study shows co-localization of activated microglia and Aβ plaques. For each cohort, we also observed eNOS both outside of and within neurons. We report below our quantitative analysis of the histological images.

In addition, our ECoG measurements recapitulated our earlier results (Bobola et al. [21], their Figure 4): we observed a strong band of 40 Hz brain activity in the left hippocampus of our treated mice, confirming FUS induction of neural activity within the brain at 40 Haz, which, as shown by Bobola et al. [21], is necessary to activate microglia.

### 3.2. Analysis of Aβ Plaque and eNOS Burdens

#### 3.2.1. Results for Younger AD Mice Treated with FUS for Three Days

**Figure 4:**
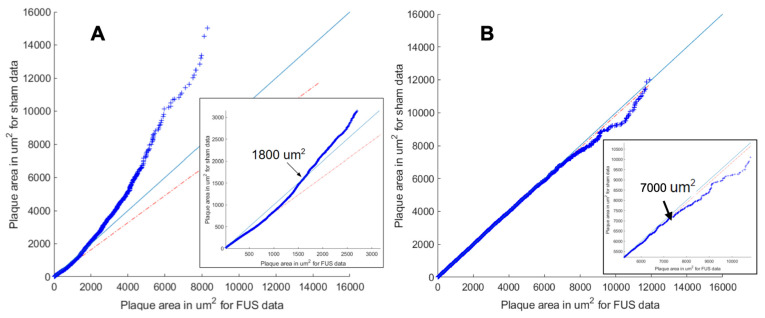
Quantile–quantile plots of Aβ plaque size (**A**) after three days of FUS versus sham FUS treatment of younger AD mice and (**B**) after five days of FUS versus sham FUS treatment of older AD mice. The blue line lies along a 45-degree angle, while the dash/dotted red line lies tangent to the lowest range of data values. The inset shows a magnified view of the point of deviation of the data from the red line.

Our quantile–quantile plots for Aβ plaques for the younger AD mice suggested that there may exist meaningful differences in Aβ plaque burden, along with their interaction with microglia, for Aβ plaque areas greater than versus less than 1800 µm^2^ (Figure 4A).

**Figure 5:**
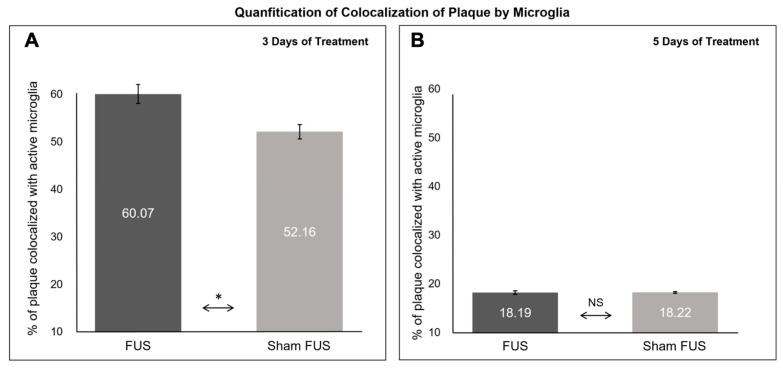
Co-localization of Aβ plaques with at least one active microglia. (**A**) Younger AD mice (sham-)treated with FUS for three days; *p*-value = 0.034. FUS % = 60.07 +/− 3.02; sham % = 52.16 +/− 3.96. (**B**) Older AD mice (sham-)treated with FUS for five days; *p*-value = 0.86. FUS % 18.19 +/− 0.78; sham % 18.22 +/− 0.34. Welch two-sample *t*-test, n = 3 mice with two histological samples per mouse for each of (sham-)treated younger and older AD mice. Here * indicates *p* < 0.01, and ‘NS’ means ‘not significant’.

The QQ plot in Figure 4A motivated sub-analysis of Aβ plaque burden and co-localization within those ranges. For example, the number of Aβ plaques (scaled by total plaque number) of all sizes co-localized with active microglia in younger mice treated with FUS was significantly greater than for sham-treated younger mice (Figure 5A). This result did not depend qualitatively upon varying the cut-off value for plaque area that we chose (1800 µm^2^) by +/− 20 percent.

**Figure 6:**
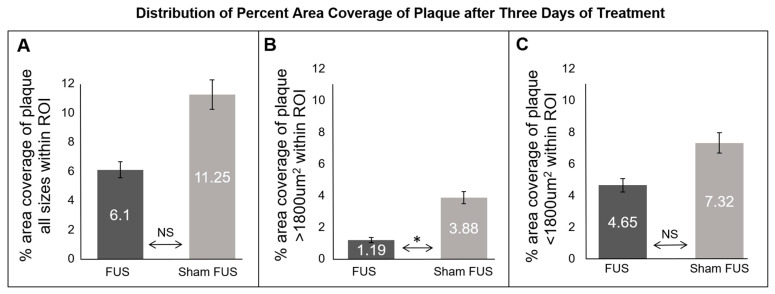
Aβ plaque burden of all sizes for younger AD mice treated with FUS versus sham FUS for 3 days. (**A**) For the percentage area of brain covered by Aβ plaque of all sizes, Welch two-sample *t*-test *p*-value = 0.10. FUS % all plaques: 6.10 +/− 1.10; sham % all plaques: 11.25 +/− 2.04. (**B**) For percent area coverage of Aβ plaques greater than 1800 µm^2^, *p*-value = 0.045. FUS % plaques > 1800 µm^2^: 1.19 +/− 0.34; sham % plaques > 1800 µm^2^: 3.88 +/− 0.76. (**C**) For the percentage area of brain covered by Aβ plaques of less than 1800 µm^2^, *p*-value = 0.18. FUS % plaques < 1800 µm^2^: 4.65 +/− 0.84; sham % plaques < 1800 µm^2^: 7.32 +/− 1.29. n = 3 mice per sham and treated cohorts with two histological samples per mouse. Here * indicates *p* < 0.01, and ‘NS’ means ‘not significant’.

The difference in the total area covered by Aβ plaques across all sizes for (sham) FUS treatment trended towards statistical significance (Figure 6A) for three-day, younger AD mice. FUS treatment significantly reduced the percentage of brain covered by Aβ plaques with plaque sizes greater than 1800 µm^2^ (Figure 6B), though with only a trend towards reducing Aβ plaques with sizes less than 1800 µm^2^ (Figure 6C).

**Figure 7:**
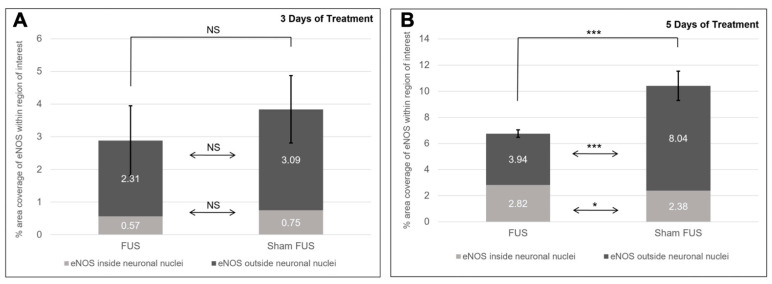
Distribution of eNOS within ROIs of AD mice. (**A**) Younger AD mice (sham-)treated for three days with FUS. *p*-Value of Welch two-sample *t*-test = 0.57 comparing percent eNOS outside neuronal nuclei. *p*-Value of Welch two-sample *t*-test = 0.06 comparing percent eNOS inside neuronal nuclei. *p*-Value for total percent area coverage of eNOS between groups = 0.59. FUS % total eNOS: 2.89 +/− 1.07; FUS % inside eNOS: 0.57 +/− 0.05; FUS % outside eNOS: 2.31 +/− 1.1. Sham % total eNOS: 3.71 +/− 1.03; sham % inside eNOS: 0.75 +/− 0.10; sham % outside eNOS: 3.09 +/− 0.96. (**B**) Older AD mice (sham-)treated for five days with FUS. Welch two-sample *t*-test = 0.001 comparing percent eNOS outside neuronal nuclei. *p*-Value of Welch two-sample *t*-test = 0.03 comparing percent eNOS inside neuronal nuclei. *p*-Value for total percent area of brain coverage of eNOS between groups = 5e-4. FUS % total eNOS: 6.73 +/− 0.31; FUS % inside eNOS: 2.82 +/− 0.29; FUS % outside eNOS: 3.94 +/− 0.52. Sham %: 10.14 +/− 0.59; sham % inside eNOS: 2.38 +/− 0.38; sham % outside eNOS: 8.04 +/− 1.4. Dark gray indicates eNOS outside of neuronal nuclei, and light gray indicates eNOS inside neuronal nuclei. Welch two-sample *t*-test, n = 3 mice with two histological samples per mouse for each cohort of (sham-)treated younger and older AD mice. Here * indicates *p* < 0.01, *** indicates *p* < 0.0001, and ‘NS’ means ‘not significant’.

The total amount of brain with eNOS showed no significant differences between sham and treated younger AD mice. In addition, the distribution of eNOS inside and outside of neuronal nuclei also did not demonstrate a difference (Figure 7A).

#### 3.2.2. Results for Older AD Mice Treated with FUS for Five Days

The quantile–quantile plots for Aβ plaques for the older AD mice reflected possible differences in Aβ plaques with area greater than 3000 µm^2^ and 7000 µm^2^ versus below that value (Figure 4B).

This motivated sub-analysis of co-localization and Aβ plaque burden above and below those ranges. The amount of brain covered by Aβ plaques of all sizes co-localized with active microglia in mice treated with actual versus sham FUS showed no statistically significant differences (Figure 5B), in contrast to what we observed for the younger AD mice. This result did not depend qualitatively upon varying the cut-off value for plaque area that we chose (3000 µm^2^ and 7000 µm^2^) by +/− 20 percent.

**Figure 8:**
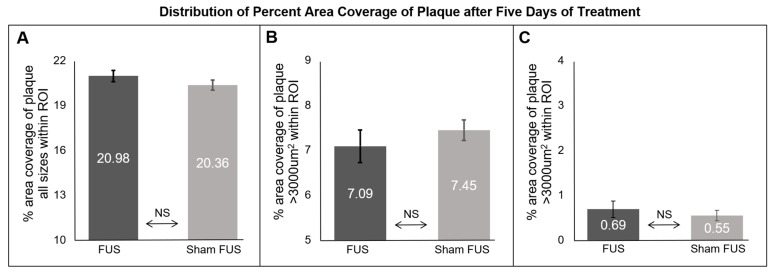
Burden of Aβ plaques in different size ranges in older AD mice (sham-)treated with 5 days of FUS. (**A**) For percent area of brain covered by Aβ plaques of all sizes, Welch two-sample *t*-test *p*-value = 0.93. FUS % all Aβ plaques: 20.98 +/− 0.75; sham % all Aβ plaques: 20.36 +/− 0.67. (**B**) For percent area of brain covered by Aβ plaques greater than 3000 µm^2^, *p*-value = 0.93. FUS % Aβ plaques > 3000 µm^2^: 7.09 +/− 0.73; sham % Aβ plaques > 3000 µm^2^: 7.45 +/− 0.46. (**C**) For percent area covered by Aβ plaques greater than 7000 µm^2^, *p*-value = 0.78. FUS % Aβ plaques < 7000 µm^2^: 0.69 +/− 0.18; sham % Aβ plaques < 7000 µm^2^: 0.55 +/− 0.12. n = 3 mice for each of the sham and treated cohorts, with two histological samples per mouse. Here ‘NS’ means ‘not significant’.

FUS treatment did not change the Aβ plaque burden in these older mice relative to sham (Figure 8).

In contrast to what we observed in the younger AD mice, however, the older AD mice treated with FUS had less total eNOS than the untreated AD mice (Figure 7B). Also, the amount of eNOS outside of neuronal nuclei was significantly less in mice treated with FUS compared with sham treatment (Figure 7B), the opposite of what we hoped to create with FUS. Interestingly, however, and unexpectedly, the distribution of eNOS within neuronal nuclei was significantly greater in mice treated with FUS than with sham FUS.

Table 1 and Table 2 compare the results for the three-day and five-day cohorts, respectively.

## 4. Discussion

Various combinations of vascular inadequacy and buildup of aberrant protein burdens occur within approximately 75% of dementia cases. The FUS protocol for one hour over five days as used by Bobola et al. [21] reduced Aβ burden in 5XFAD mice by ~50%, through activation of microglia, which has been confirmed and extended by others [22]. A different FUS protocol for one hour/day, three days per week, for three months by Eguchi et al. [23] reduced the Aβ burden by ~15% by using the same mouse model while—in a causal manner—significantly increasing cerebrovascular flow by ~50% in the same mouse model. Taken together, these observations motivated our construction of a novel FUS protocol that combined the protocols by Bobola et al. [21] and Eguchi et al. [23]—Figure 1. In this way we hoped to reduce the Aβ burden in a synergistic fashion via activation of microglia and increase in eNOS, tested here in vivo.

### 4.1. Regarding Aβ Plaque Distribution

Our results show that three days of our combination FUS protocol applied to younger mice activated their microglia such that they co-localized with Aβ plaques, relative to sham treatment. We also observed a net decrease in Aβ plaque burden for plaque sizes above 1800 µm^2^, with a trend towards reduction in smaller plaques and total plaque burden, all relative to sham treatment. In contrast, increasing the treatment to five days and applying the same protocol to significantly older 5XFAD mice did not meaningfully activate microglia nor did it reduce Aβ plaque burden, all relative to sham treatment. Table 1 and Table 2 summarize the differences in observed microglia and Aβ plaques between the younger versus older mice that we studied, for both sham and treated cohorts. The older sham mice had a nearly twofold increase in plaques relative to the younger sham mice (*p* < 0.01) but with a comparable number of microglia, consistent with observations by others [24,25]. Therefore, for the older versus younger mice, there was a much larger Aβ plaque burden for FUS to address by activating a comparable number of microglia. In addition, endogenous microglia activation over months in mice (over years in humans) leads to exhaustion and immunosenescence of the microglia, making them less efficient in attacking Aβ [31]. For the older mice, we therefore hypothesize that there simply were not enough healthy microglia available to activate via FUS and successfully reduce the substantially larger Aβ burden for the old mice relative to the younger mice.

### 4.2. Regarding eNOS Distribution

We differentiated between extra- and intra-neuronal eNOS distributions, unlike Eguchi et al. [23], who showed that more total eNOS increased net cerebrovascular flow in a causal and functionally consequential way. Table 1 and Table 2 summarize the differences in observed eNOS distribution between the younger mice versus older mice that we studied, for both sham and treated cohorts. Without treatment (the sham group), total area coverage of all eNOS for older mice was more than twice that for the younger mice. Also, the percentage of total eNOS within neuronal nuclei nearly doubled from younger to older mice, while the percentage of total eNOS associated with extra-neuronal eNOS did not change for younger versus older mice. FUS treatment of younger mice did not alter their total nor partitioned eNOS burden. However, FUS treatment of older mice produced significant changes in eNOS distribution relative to sham: a decrease in the % area coverage of total eNOS and of extra-neuronal eNOS and an increase in the % area coverage of intra-neural eNOS. The reduction in extra-neuronal eNOS is the opposite of what we intended. The results by Eguchi et al. [23] therefore suggest that our novel ultrasound protocol may decrease rather than increase cerebral blood flow relative to sham treatment, thereby exacerbating vascular dementia’s ischemic effect. Interestingly, however, Li et al. [32] demonstrated that intra-neuronal eNOS overexpression can protect against ischemic injury by enhancing BDNF secretion by the neurons, thereby reducing apoptosis and supporting synaptic plasticity. Quoting from their paper, “Our findings suggest that eNOS expressed by neurons functions as a transducer of survival signals, which is upregulated and activated by ischemic stimulation.” Thus, the effects of our observed increase in intra-neuronal eNOS generated by our FUS protocol is in potential counterbalance to the effects of reduced extra-neuronal eNOS also generated by our FUS protocol. Further amendments to the FUS protocol may optimize this potential therapeutic effect while minimizing or even reversing its potential ischemic effect. Future research that simultaneously assays eNOS distribution, synaptic function, cerebrovascular flow, and ischemia along with behavioral tests can explore this possibility.

That our results depend on the age of the mice highlights an important issue for translation of FUS-based methods of reducing the Aβ burden associated with progressing Alzheimer’s Disease. As people age, their Aβ burden increases substantially [33]. Mouse-based studies of FUS for treating AD (and aging in general) should ensure the use of sufficiently older mice—those known to have representative levels of brain-tissue pathology and brain dysfunction—as have Götz and colleagues [34], for example. In addition, recall the human results obtained by Nicodemus et al. [20]—who, along with Eguchi et al. [23] in their in vivo study, showed changes in net cerebrovascular flow created by ultrasound—and mouse-based studies showing FUS-induced changes in synaptic function, as reviewed in Balbi et al. [19]. Future in vivo and human studies should therefore look beyond changes in aberrant protein burden generated by FUS—and do so in appropriately older recipients of FUS—to fully appreciate its possible therapeutic effects. Finally, our findings also identify two candidate therapeutic windows for our FUS treatment of AD: earlier intervention to maximize Aβ plaque removal via activation of microglia and later intervention to yield synaptic protection if it is possible to mitigate the potential ischemic risk caused by the differential effects of FUS on eNOS.

## 5. Limitations

Our original power analysis, based on our earlier published work, supported our use of n = 3 mice in each of the sham and treated cohorts. We did not achieve the same results, however. Post hoc power analysis with G*power, as reported in Section 2 (our Methods Section), and using our results for total eNOS distribution for the three-day younger cohort (Figure 7A) show that we would need to use n = 7 mice to resolve a possible difference between sham and treated cohorts. In contrast, power analysis with G*power as per our Methods Section and using our results for total Aβ plaque based on the five-day older cohort (Figure 8) show that we would need to use over three hundred mice to see a possible difference between the sham and treated cohorts, though significantly fewer mice for assessing the effect of FUS on the largest Aβ plaque for the five-day older cohort. Therefore, future studies with more mice would likely demonstrate meaningful differences in the effect of FUS on eNOS distribution versus age, and possibly Aβ plaque burden, relative to what we reported here. Likely also important would be increasing the FUS intensity—we chose a value less than half of that in Bobola et al.’s study [21]. Additionally, we focused on global distributions of Aβ plaques, as did the studies that motivated our work [21,23]. We did not separately assay parenchymal versus vascular amyloid burdens, however, an important topic for future work. Finally, we chose to use only female 5XFAD mice, because of our parallel research with another AD mouse model (3xTg-AD mice) that is exclusively female. Female 5XFAD mice manifest more pathology than their male counterparts—future work should address this difference.

Auditory stimulation by FUS can activate brain along with or, possibly, instead of direct activation of neurons by FUS energy effects [35]. We did not check for auditory effects in the present study because we relied upon our previous research in the same laboratory, for which we determined that there were no auditory differences between sham and treated conditions along with differential focal effects between hemispheres of FUS consistent with the placement of their FUS focus (Bobola et al. [21]).

## 6. Conclusions

Pure Alzheimer’s Disease, vascular dementia, and their combination contribute to the majority of dementia cases. AD arises in part due to the buildup of and associated damage created by aberrant proteins (Aβ, Tau); vascular dementia is caused by reduced cerebral blood flow, which induces ischemic damage. Our results demonstrate that a large difference in the number of total Aβ plaques as a function of age combined with negligible differences in available microglia can play an important role in the ability of FUS to activate the latter in order to help clear the former. Thus, with support of future work, our FUS protocol may one day find value in reducing the early buildup of Aβ in dementia patients. Our results also show that FUS can manipulate intra-neuronal as well as extra-neuronal processes—here eNOS distribution—with thus-far unknown countervailing effects: possible synaptic protection versus possible decreased cerebral blood flow, hence ischemia. Future research should resolve these differential effects, as well as assaying FUS’s possible effects on vascular amyloid burden. If successful, such research may one day identify an FUS protocol as a promising therapy for vascular or for mixed dementia during later stages of these diseases.

## Figures and Tables

**Table 1:** Comparison of phenotypic values (sham) and associated FUS treatment values for younger mice treated for three days. All quantities except microglia counts are reported as percentages of the area of ROI. Total and activated microglia counts are reported as the number of microglia per µm^2^. Here, * indicates *p* < 0.01.

Three Days of Treatment			
(n = 3 per Group)			
(Median +/−) SE			
Age 7.5 +/− 0.51 (Months)			
Parameter	FUS		Sham FUS
% plaque burden	6.10 +/− 1.09		11.25 +/− 2.04
# of microglia/um^2^	0.18 +/− 0.07	*	0.15 +/− 0.11
# activated microglia/um^2^	0.10 +/− 0.10		0.09 +/− 0.09
% co-localized plaque	60.08 +/− 3.02		52.17 +/− 3.96
% area eNOS	2.89 +/− 1.07		3.71 +/− 1.03
% eNOS outside neuronal nuclei	90.16 +/− 4.77		89.63 +/− 1.27
% eNOS inside neuronal nuclei	9.84 +/− 4.77		10.37 +/− 1.27

**Table 2:** Comparison of phenotypic values (sham) and associated FUS treatment values for older mice treated for five days. All quantities except microglia counts are reported as percentages of the area of ROI. Total and activated microglia count are reported as the number of microglia per µm^2^. Here, * indicates *p* < 0.01, ** indicates *p* < 0.001, and *** indicates *p* < 0.0001.

Five Days of Treatment			
(n = 3 per Group)			
(Median +/− SE)			
Age 12.17 +/− 0.13 (Months)			
Parameter	FUS		Sham FUS
% plaque burden	20.98 +/− 0.75		20.35 +/− 0.67
# of microglia/um^2^	0.05 +/− 0.03		0.05 +/− 0.01
# activated microglia/um^2^	0.03 +/− 0.03		0.03 +/− 0.03
% co-localized plaque	18.19 +/− 0.78		18.22 +/− 0.33
% area eNOS	6.73 +/− 0.31	***	10.14 +/− 0.59
% eNOS outside neuronal nuclei	64.63 +/− 0.99	**	80.30 +/− 1.89
% eNOS inside neuronal nuclei	35.50 +/− 0.99	*	19.89 +/− 1.91

## Data Availability

Original data available at Zenodo: https://doi.org/10.5281/zenodo.21187946.
